# Comparison of placental growth factor and fetal flow Doppler ultrasonography to identify fetal adverse outcomes in women with hypertensive disorders of pregnancy: an observational study

**DOI:** 10.1186/1471-2393-13-161

**Published:** 2013-08-13

**Authors:** Attila Molvarec, Nóra Gullai, Balázs Stenczer, Gergely Fügedi, Bálint Nagy, János Rigó Jr

**Affiliations:** 1 First Department of Obstetrics and Gynecology, Semmelweis University, Baross utca 27, Budapest H-1088, Hungary

**Keywords:** Hypertension, Placental growth factor, PlGF, Preeclampsia, IUGR, Doppler, Growth restriction

## Abstract

**Background:**

Hypertensive disorders of pregnancy and intrauterine growth restriction (IUGR) are leading causes of maternal and perinatal morbidity and mortality. Failure to detect intrauterine growth restriction in women at high risk has been highlighted as a significant avoidable cause of serious fetal outcome. In this observational study we compare fetal flow using Doppler ultrasonography with a new test for placental growth factor (PlGF) to predict fetal adverse events.

**Methods:**

Eighty-nine women with hypertensive disorders of pregnancy (24 with chronic hypertension, 17 with gestational hypertension, 12 with HELLP syndrome, 19 with preeclampsia and 17 with superimposed preeclampsia) were enrolled. A single maternal blood sample to measure free PlGF (Alere Triage) taken before 35 weeks of pregnancy was compared to the last Doppler ultrasound measurement of fetal flow before delivery. PlGF was classified as normal (PlGF≥100 pg/ml), low (12<PlGF<100) or very low (PlGF≤12 pg/ml). A positive test for abnormal fetal flow was defined as either signs of centralisation of the fetal circulation or diastolic block or reverse flow in the umbilical artery or descending aorta; this was a criterion for delivery. Fetal outcomes were intrauterine growth restriction and birth before 37 weeks of pregnancy.

**Results:**

In total 61/89 women had a preterm birth and 22 infants had IUGR. Of those who delivered preterm, 20/20 women with abnormal fetal flow and 36/41 (87.8%) women with normal fetal flow had low or very low PlGF. Of those infants with IUGR, 22/22 had low or very low maternal PlGF and 10/22 had abnormal fetal flow.

**Conclusions:**

PlGF may provide useful information before 35^th^ gestational week to identify fetuses requiring urgent delivery, and those at risk of later adverse outcomes not identified by fetal flow Doppler ultrasonography.

## Background

Hypertensive disorders are one of the most common complications of pregnancy, with a prevalence of 6–22% [1,2]. These conditions are responsible for the majority of maternal and perinatal morbidity and mortality, including intrauterine growth restriction (IUGR). A recent study has shown that unrecognised IUGR is the single largest risk factor to pregnancies that end in stillbirth [3]. Research focus is now on improving antenatal detection of fetuses at risk, to allow selective management, timely delivery and minimisation of serious outcomes.

Placental growth factor (PlGF) is a member of the vascular endothelial growth factor (VEGF) family. It is produced mainly by the placenta, and has potent pro-angiogenic effects. In normal uncomplicated pregnancy, PlGF levels rise until approximately pregnancy week 32 and then fall until delivery [4]. In pregnancies complicated by preeclampsia before the 37^th^ week with or without IUGR, PlGF levels are significantly lower [5].

The role of determination of sFlt-1, PlGF and other angiogenic factor levels in maternal peripheral blood in the prediction and diagnosis of preeclampsia has been extensively studied in recent years [6-20]. We recently added to the limited information available about the levels of these factors in other forms of hypertension in pregnancy [21-24] and found that free PlGF measured before 35 weeks of pregnancy may predict preterm delivery in all forms of hypertensive disorders of pregnancy [25]. There is still little information to date on their ability to predict fetal outcomes such as intrauterine growth restriction through their assessment of placental function [26].

In this study, we used a new method, the Alere Triage® PlGF test, for measuring free PlGF levels in the peripheral blood of hypertensive pregnant women before the 35^th^ gestational week of pregnancy. We examined its prognostic efficiency regarding adverse fetal outcomes in all forms of hypertensive disorders of pregnancy, compared with currently recommended assessment of fetal wellbeing.

## Methods

In this observational study, 89 women with hypertensive disorders of pregnancy (19 women with preeclampsia, 12 with hemolysis, elevated liver enzymes and low platelet count (HELLP) syndrome, 17 with superimposed preeclampsia (SIPE), 24 with chronic hypertension, and 17 with gestational hypertension) were enrolled. The study subjects were selected from the previously reported groups of hypertensive pregnant women [25] based on availability of Doppler ultrasound results. All subjects were Caucasian and resided in the same geographic area. Blood samples were taken in the First Department of Obstetrics and Gynecology, Semmelweis University, Budapest, Hungary between May 2008 and October 2010. The blood draw occurred between the 22^nd^ and 34^th^ completed gestational week at the time of the first routine clinical blood test and repeated only if women were reclassified. An interview was carried out with every subject after the diagnosis and again 12 weeks after the delivery, and if necessary, reclassification was done independently of the PlGF level. All subjects gave written informed consent prior to the involvement in the study. The study protocol was approved by the Regional and Institutional Committee of Science and Research Ethics of the Semmelweis University (TUKEB 52/2008). The study was conducted in accordance with the Declaration of Helsinki.

Hypertension was diagnosed if systolic blood pressure (BP) was ≥140 mmHg, or diastolic BP ≥90 mmHg, on two occasions, six hours apart. According to the definitions of the ACOG and NHBPEP we classified the subjects’ diagnoses into four groups: chronic hypertension, gestational hypertension, preeclampsia, and superimposed preeclampsia. We separately categorised any women with hypertension, proteinuria and the syndrome of hemolysis, elevated liver enzymes and low platelet count as HELLP.

Chronic hypertension was diagnosed if high blood pressure developed prior to pregnancy or before the 20^th^ week of gestation, or if hypertension persisted for more than 12 weeks postpartum. Gestational hypertension was applied to women who developed new-onset hypertension after the 20^th^ week of gestation, in the absence of proteinuria, confirmed after delivery. Patients who later progressed to preeclampsia were excluded from this group, and following repeated blood draw, they were included in the pre-eclamptic group. Preeclampsia was defined as hypertension and proteinuria (≥ 300 mg / 24 hours, or ≥+ by urine dipstick) with an onset after the 20^th^ gestational week. Severe preeclampsia was diagnosed if one of the following occurred: systolic BP ≥160 mmHg, diastolic BP ≥110 mmHg, proteinuria ≥5000 mg per 24 hours or ≥+++ by urine dipstick, partial HELLP syndrome, signs of renal insufficiency, pulmonary edema or threatening eclampsia. The diagnosis of HELLP syndrome was made based on characteristic laboratory findings (platelet count <150 G/l, SGOT and SGPT>70 U/l, LDH>600 U/l). All of the subjects in this group also met the criteria for severe preeclampsia. Subjects with chronic hypertension who developed proteinuria after 20 weeks of gestation were categorised as superimposed preeclampsia. Subjects with preeclampsia, superimposed preeclampsia or HELLP syndrome were further grouped into early-onset (disease onset before 34^th^ completed gestational week). Women with multiple gestations were not enrolled in this study.

A newborn was considered small for gestational age (SGA) if the birth weight was below the 10^th^ percentile for gestational age and gender, according to a Hungarian birth weight percentile table. All neonates with SGA had an asymmetric size (normal length, but low weight for gestational age at delivery), indicating that they had intrauterine growth restriction and were not constitutionally small. In addition, none of them had fetal structural abnormalities or genetic diseases. For the determination of the abnormalities of fetal circulation we evaluated the last Doppler ultrasound results prior to delivery. Blood flow was examined in the umbilical and in the fetal middle cerebral arteries, as well as in the descending aorta of the fetus. Abnormal fetal flow was diagnosed if diastolic block or reverse flow in the umbilical artery or descending aorta, or if signs of the centralisation of the fetal circulation (increased resistance in the umbilical artery and/or descending aorta with decreased resistance in the middle cerebral artery) were present.

Our protocol for delivery dictates birth by Cesarean section, as clinically required, for women at any gestational age with HELLP syndrome, severe uncontrollable hypertension, severe proteinuria, with signs of renal insufficiency, pulmonary edema, threatening eclampsia (persistent headache and visual disturbance), low platelet count (< 100 000 /μl), elevated liver enzymes (SGOT>70 U/l) with epigastric pain, or evidence of fetal compromise (abnormal fetal flow or pathological CTG), severe IUGR or severe oligohydramnios. In the absence of any of these factors, we continue conservative management until the 37^th^ completed gestational week.

After each blood draw, the EDTA-anticoagulated plasma samples were immediately centrifuged at 3000 g for 10 min at 4°C and the supernatants were kept frozen at −80°C until assayed. Plasma was analysed for free PlGF using the Alere Triage® PlGF assay according to the manufacturer’s instructions. Using fluorescently-labelled monoclonal antibodies against PlGF for PlGF quantification, this immunoassay is run with a single-use disposable plastic assay test cartridge in conjunction with the Triage Meter. Briefly, 250 microliter of thawed plasma (room temperature) is pipetted into the sample port of a new test cartridge. The cartridge is inserted into the meter and results are displayed in approximately 15 minutes in pg/ml. The cartridge contains chemistries for on-board positive and negative control systems. Control systems at the level of the cartridge and meter ensure that the quantitative PlGF result is valid. Calibration information is supplied by the manufacturer in the form of a lot-specific EPROM chip that is contained within each kit of devices. The measurable range of the assay is 12–3000 pg/ml. Concentrations below 12 pg/ml are value assigned based on the calibration curve, but this value is displayed to the user as a qualitative result “<12 pg/ml”. Women were tested up to 34 completed weeks, as recommended in the manufacturer’s product insert. The intra/ inter-assay coefficients of variation at mean concentrations of 85.2 and 1300 pg/ml were 12.1/12.8% and 11.7/13.2%, respectively.

Although PlGF concentrations fluctuate during pregnancy [4,27], original cut-off levels based on the 5^th^ percentile of a PlGF level in a normal healthy pregnant population do not differ significantly from an absolute threshold of 100 pg/ml in women presenting with signs and symptoms of preeclampsia before the 35^th^ week of pregnancy [Chappell et al., personal communication, submitted for publication, see Appendix for full list of researchers]. Therefore levels were classified as normal (PlGF≥100 pg/ml), low (12 pg/ml<PlGF<100 pg/ml) or very low (PlGF≤12 pg/ml).

Descriptive statistics were used to present the clinical characteristics. Outcomes were broken out into three PlGF groups and abnormal/ normal fetal flow, as described above. All statistical analyses were conducted using the MATLAB version 8.0. P-values were calculated using a two-tailed Fisher Exact test of each 2 × 2 contingency table (in this case a single PlGF cut-off of 100 pg/ml was used).

## Results

In total 89 women with hypertensive disorders of pregnancy were recruited into the study. The demographics and clinical characteristics of the study participants are presented in Table 1.

**Table 1:** Demographics and clinical characteristics (N=89)

**Diagnosis**	**All (N=89)**	**GHT (n=17)**	**CHT (n=24)**	**PE (n=19)**	**HELLP (n=12)**	**SIPE (n=17)**
**Maternal age (years)**	33 (30–36)	34 (33–35)	33 (30–37)	33 (27–37)	30 (29–33)	34 (32–36)
**Pre-gestational BMI (kg/m**^**2**^**)**	26.2 (23.1–33.1)	26.2 (23.1–30.8)	30 (25.9–36.2)	25.6 (22.4–29.7)	29.8 (23.7–35.5)	24.4 (21.9–30)
**Primiparous women (%)**	51 (57.3)	11 (64.7)	12 (50)	11 (57.9)	9 (75)	8 (47.1)
**Current smoking (%)**	7 (7.9)	0 (0)	2 (8.3)	1 (5.3)	2 (16.7)	2 (11.8)
**Previous history of PE (%)**	12 (13.5)	1 (5.9)	3 (12.5)	3 (15.8)	2 (16.7)	3 (17.6)
**GA at blood draw (weeks)**	32 (28–33)	33 (31–34)	33 (31–34)	30 (28–33)	29 (27–32)	31 (28–32)
**GA at delivery (weeks)**	34 (30–37)	38 (36–39)	37 (34–38)	31 (28–34)	29 (27–32)	32 (30–36)
**Blood draw to delivery time interval (days)**	8 (1–29)	29 (24–51)	20 (4–43)	1 (0–3)	1 (0–2)	8 (3–25)
**Fetal birth weight (grams)**	1900 (1150–3100)	3330 (2690–3860)	2700 (2105–3360)	1220 (990–1770)	1125 (845–1420)	1750 (1100–2560)
**Fetal birth length (cms)**	45 (39–52)	51 (48–55)	51 (45–53)	40 (37–45)	39 (36–44)	45 (40–51)
**Preterm birth (%)**	61 (68.5)	6 (35.3)	11 (45.8)	18 (94.7)	12 (100)	14 (82.4)
**IUGR (%)**	22 (24.7)	3 (17.6)	2 (8.3)	8 (42.1)	4 (33.3)	5 (29.4)
**Early-onset PE (%)**	44 (49.4)	0 (0)	0 (0)	17 (89.5)	11 (91.7)	16 (94.1)
**Severe PE (%)**	41 (46.1)	0 (0)	0 (0)	18 (94.7)	12 (100)	11 (64.7)
**C-Section (%)**	78 (87.6)	12 (70.6)	19 (79.2)	19 (100)	12 (100)	16 (94.1)

Continuous variables are reported as Median (IQR) and categorical variables are reported as n (%), where percent is relative to the column total. Gestational hypertension (GHT); chronic hypertension (CHT); preeclampsia (PE); hemolysis, elevated liver enzymes and low platelet count syndrome (HELLP); superimposed preeclampsia (SIPE); body mass index (BMI); gestational age (GA); intrauterine growth restriction (IUGR); Cesarean section (C-section).

By outcome, 61/89 women had a preterm birth and 22 neonates had IUGR. Table 2 shows the results of the fetal flow and PlGF tests by outcome (preterm birth and IUGR) and by diagnoses. All 20 women with abnormal fetal flow had a PlGF<100 pg/ml so that the overall concordance between fetal flow and PlGF was high (p-value=0.0023, two tailed Fisher Exact test of the contingency table dividing the 89 women by PlGF and fetal flow).

**Table 2:** Results of PlGF and fetal flow tests, by outcome and diagnosis (N=89)

	**Normal PlGF**		**Low PlGF**		**Very Low PlGF**	
**Fetal flow**	**Normal**	**Abnormal**	**Normal**	**Abnormal**	**Normal**	**Abnormal**
**n**	22	0	23	3	24	17
**Percent of N**	(24.7)	0.0	(25.8)	(3.4)	(27.0)	(19.1)
**Preterm**	5 (22.7)		14 (60.9)	3 (100.0)	22 (91.7)	17 (100.0)
**IUGR**	0 (0.0)		3 (13.0)	3 (100.0)	9 (37.5)	7 (41.2)
**CHT**	8 (36.4)		11 (47.8)	0 (0.0)	2 (8.3)	3 (17.6)
**GHT**	10 (45.5)		3 (13.0)	1 (33.3)	2 (8.3)	1 (5.9)
**HELLP**	0 (0.0)		2 (8.7)	0 (0.0)	6 (25.0)	4 (23.5)
**PE**	1 (4.5)		2 (8.7)	1 (33.3)	10 (41.7)	5 (29.4)
**SIPE**	3 (13.6)		5 (21.7)	1 (33.3)	4 (16.7)	4 (23.5)

Breakout of N=89 subjects by three placental growth factor test result groups (normal PlGF≥100, low 12<PlGF<100, and very low PlGF≤12) and two fetal flow groups (abnormal, normal). The breakout by outcomes (preterm birth, IUGR infant) and diagnosis shows the number and percent (relative to n) for each category. Intrauterine growth restriction (IUGR); chronic hypertension (CHT); gestational hypertension (GHT); hemolysis, elevated liver enzymes and low platelet count syndrome (HELLP); preeclampsia (PE); superimposed preeclampsia (SIPE).

Considering the PlGF test results for the 28 women who had both a term birth and an infant of normal birth weight, 1/1 woman with preeclampsia, 2/3 women with SIPE, 9/11 women with GHT and 5/13 women with CHT had a normal PlGF result. All of these women had a normal fetal flow.

Table 3 shows PlGF test results in women with normal and abnormal fetal flow. Of those who delivered preterm, 20/20 women with abnormal fetal flow and 36/41 women with normal fetal flow had low or very low PlGF. Five women who had a preterm delivery had both a normal fetal flow and a normal PlGF test; of these five, 2 women had an early delivery because of premature rupture of membranes. All 5 women had infants with a normal birth weight. Of the IUGR neonates, 22/22 had low or very low maternal PlGF and 10/22 had abnormal fetal flow. In the subset of 69 women who had normal fetal flow, a positive PlGF test result was significantly associated with adverse fetal outcomes (p-value<0.0001 for preterm delivery and p-value=0.0069 for IUGR).

**Table 3:** Adverse fetal outcomes by fetal flow (abnormal, normal) and PlGF (normal, low, very low) results (N=89)

	**Abnormal fetal flow**			**Normal fetal flow**		
**PlGF**	**Very Low**	**Low**	**Normal**	**Very Low**	**Low**	**Normal**
**n**	17	3	0	24	23	22
**Percent of N**	19.1	3.4	0.0	27.0	25.8	24.7
**Preterm**	17 (100.0)	3 (100.0)		22 (91.7)	14 (60.9)	5 (22.7)
**p-value**				**<0.0001**		
**IUGR**	7 (41.2)	3 (100.0)		9 (37.5)	3 (13.0)	0 (0.0)
**p-value**				**0.0069**		

Breakout of N=89 subjects by three placental growth factor groups (normal is PlGF≥100, low is 12<PlGF<100, and very low is PlGF≤12) and fetal flow (abnormal vs normal). The breakout by preterm delivery and IUGR shows the number and percent (relative to n) for each category. The p-values are from a two-tailed Fisher Exact test of the 2×2 contingency table (with PlGF Low and Very Low grouped together) for each outcome (preterm and IUGR) in the subset with normal fetal flow.

Twenty-four of our study participants had pathological CTG, 21 of whom had low or very low PlGF. Oligohydramnios occurred in 27 cases, 20 of them had low or very low PlGF. Of those who delivered preterm, 19/20 women with pathological CTG and 37/41 women with normal CTG had low or very low PlGF. In the preterm delivery group, 19/21 women with oligohydramnios and 37/40 women with normal amniotic fluid volume had low or very low PlGF. In the subset of 41 women with preterm delivery and normal fetal flow, 10/11 women with pathological CTG and 11/13 women with oligohydramnios had low or very low PlGF. Of the 22 IUGR neonates, 10 had pathological CTG and 10 had oligohydramnios.

## Discussion

This study aimed to assess the performance of the Alere Triage® PlGF test, a rapid test for measuring PlGF levels in the maternal peripheral blood. Our study population consisted of women with chronic hypertension, gestational hypertension, HELLP syndrome, preeclampsia and superimposed preeclampsia. We earlier evaluated the diagnostic accuracy of this test in the study groups and healthy pregnant control subjects [25] and found that using a gestational age-dependent threshold of 5% of a normal population, a positive PlGF test predicted delivery before 37 weeks in over 90% of hypertensive women. In this study we compared the diagnostic value of PlGF measured before 35 weeks using two absolute thresholds with the last Doppler ultrasonography before delivery in identifying preterm birth and IUGR neonates.

We found that PlGF concentration below 100 pg/ml identified all women with hypertensive disorders of pregnancy who required urgent delivery following an abnormal fetal flow result, and predicted all IUGR neonates, and 56/61 women who had a preterm delivery. Nearly 60% of women with normal fetal flow results had a preterm delivery. Our protocol for delivery dictated birth by Cesarean section, as clinically required, for women at any gestational age with evidence of fetal compromise including abnormal fetal flow, pathological CTG and severe oligohydramnios. The Triage PlGF test identified the majority of women with normal fetal flow who needed to be delivered preterm due to pathological CTG or oligohydramnios. Considering both test results together, no woman with a normal fetal flow and normal PlGF had an IUGR infant, and only 5 had a preterm delivery, 2 of whom were due to premature rupture of membranes.

In a small study Benton et al. [28] found that Triage PlGF differentiated placental IUGR from constitutionally small fetuses with a sensitivity of 100% and a specificity of 86%. Consistently, all of our SGA neonates were asymmetrically small with no fetal structural abnormalities or genetic diseases suggesting placental IUGR and had low or very low PlGF. In 2007 Schlembach et al. [29] correlated levels of angiogenic growth factors, including PlGF, with Doppler ultrasound parameters in women with preeclampsia and intrauterine growth restriction (suspected by ultrasound measurements and confirmed by birth weight). Maternal levels of PlGF were inversely correlated with PI values in both the umbilical and uterine arteries. PlGF levels in the umbilical vein were below the detection limit in nearly all samples of IUGR fetuses and lower than in those with preeclampsia (p<0.001). In our study, we observed low or very low PlGF even in hypertensive women with normal fetal flow and an IUGR neonate.

Taylor et al. reported in 2003 that maternal circulating PlGF levels in preeclampsia are lower if accompanied by IUGR [11]. Furthermore they showed that in normotensive subjects with IUGR, PlGF concentrations are also decreased compared to controls. Our knowledge about IUGR in chronic and gestational hypertension in this regard is deficient. We found a positive PlGF test in all women with an abnormal fetal flow, all women with an IUGR neonate, the majority of women with pathological CTG or oligohydramnios, as well as in a high proportion of women carrying normal-size fetuses, many of whom had preterm delivery. Thus, the sensitivity of the PlGF test for fetal risk (IUGR, abnormal fetal flow, pathological CTG, oligohydramnios, preterm delivery) in the hypertension group with and without proteinuria was excellent.

The potential clinical impact of these findings is that PlGF may provide useful information before 35^th^ gestational week to identify fetuses requiring urgent delivery, and those at risk of later adverse outcomes not identified by fetal flow Doppler ultrasonography. Similarly, for all women with hypertensive disorders of pregnancy, a combination of a normal fetal flow and normal PlGF test may identify women at lower risk of adverse outcomes.

However, the weakness of our study is its retrospective observational design following normal clinical practice and the low case number. Additionally, we did not measure circulating levels of anti-angiogenic factors (sFlt-1, soluble endoglin). In this retrospective observational study of a specific target population we are unable to calculate the specificity of either test with respect to preterm delivery or IUGR infant, or to calculate negative or positive predictive values. There is a need for prospective studies to prove the safety and efficiency of the test in the clinical management of hypertension in pregnancy.

## Conclusions

The Triage test is a quick reliable method for measuring circulating levels of free PlGF. The test may provide useful information before 35^th^ gestational week to identify fetuses requiring urgent delivery, and those at risk of later adverse outcomes not identified by fetal flow Doppler ultrasonography.

### Ethical approval

The study protocol was approved by the Regional and Institutional Committee of Science and Research Ethics of the Semmelweis University, Budapest, Hungary (TUKEB 52/2008).

## Appendix

### Personal communication from the following researchers

Lucy C Chappell, PhD; Suzy Duckworth, MBBS; Paul T Seed, CStat; Melanie Griffin, MBChB; Lucy Mackillop, MA; Nigel Simpson, MBBS; Jason Waugh, MBBS; Dilly Anumba, MD; Louise C Kenny, PhD; Christopher W Redman, MBCChir; Andrew H Shennan, MD.

## Abbreviations

(NHBPEP): National High Blood Pressure Education Program Working Group on High Blood Pressure in Pregnancy; (ACOG): American Congress of Obstetricians and Gynecologists; (PlGF): Placental growth factor; (VEGF): Vascular endothelial growth factor; (sFlt-1): Soluble fms-like tyrosine kinase-1; (HELLP): Hemolysis, elevated liver enzymes and low platelet count syndrome; (SGA): Small for gestational age; (IUGR): Intrauterine growth restriction; (SIPE): Superimposed preeclampsia; (SGOT): Serum glutamic oxaloacetic transaminase; (SGPT): Serum glutamic pyruvic transaminase; (CTG): Cardiotocography; (EDTA): Ethylenediaminetetraacetic acid.

## Competing interests

The authors declare that they have no competing interests.

## Authors’ contributions

AM conceived of the study, participated in its design and coordination, performed statistical analyses and drafted the manuscript. NG, BS and GF collected biological samples, acquired clinical data and performed PlGF measurements. BN and JR participated in the design and coordination of the study. All authors read and approved the final manuscript.

## Pre-publication history

The pre-publication history for this paper can be accessed here:

http://www.biomedcentral.com/1471-2393/13/161/prepub
